# Brain-Targeted Intranasal Delivery of Zotepine Microemulsion: Pharmacokinetics and Pharmacodynamics

**DOI:** 10.3390/pharmaceutics14050978

**Published:** 2022-04-30

**Authors:** Sravanthi Reddy Pailla, Sunitha Sampathi, Vijayabhaskarreddy Junnuthula, Sravya Maddukuri, Sujatha Dodoala, Sathish Dyawanapelly

**Affiliations:** 1 Department of Pharmaceutics, National Institute of Pharmaceutical Education and Research (NIPER), Hyderabad 500037, India; sravyreddy.pailla@gmail.com; 2 GITAM School of Pharmacy, GITAM Deemed to be University, Hyderabad 502329, India; smadduku@gitam.edu; 3 Drug Research Program, Faculty of Pharmacy, University of Helsinki, Viikinkaari 5 E, 00790 Helsinki, Finland; 4 Institute of Pharmaceutical Technology, Sri Padmavati Mahila Visvavidyalayam, Tirupati 517502, India; drsujathasai@gmail.com; 5 Department of Pharmaceutical Science and Technology, Institute of Chemical Technology, Mumbai 400019, India

**Keywords:** brain targeting, catalepsy, zotepine, nose-to-brain delivery, pharmacokinetics, microemulsion

## Abstract

The purpose of our study was to improve the solubility, bioavailability, and efficacy of zotepine (ZTP) by brain-targeted intranasal delivery of microemulsion (ME) and its physicochemical properties, the pharmacokinetic and pharmacodynamic parameters were evaluated. The optimized ME formulations contain 10% *w/w* of oil (Capmul MCM C8, monoglycerides, and diglycerides of caprylic acid), 50% *w/w* of S_mix_ (Labrasol and Transcutol HP, and 40% *w/w* of water resulting in a globule size of 124.6 ± 3.52 nm with low polydispersity index (PDI) (0.212 ± 0.013) and 2.8-fold higher permeation coefficient through porcine nasal mucosa compared to pure drug). In vitro cell line studies on RPMI 2650, Beas-2B, and Neuro-2A revealed ZTP-ME as safe. ZTP-ME administered intranasally showed higher AUC_0–t24_ (18.63 ± 1.33 h × µg/g) in the brain by approximately 4.3-fold than oral ME (4.30 ± 0.92 h × µg/g) and 7.7-fold than intravenous drug solutions (2.40 ± 0.36 h × µg/g). In vivo anti-schizophrenic activity was conducted using catalepsy test scores, the formulation showed better efficacy via the intranasal route; furthermore, there was no inflammation or hemorrhage in the nasal cavity. The results concluded that the ZTP microemulsion as a safe and effective strategy could greatly enhance brain distribution by intranasal administration.

## 1. Introduction

Schizophrenia is a chronic, complex persistent health disorder caused by neurotransmission abnormalities. The hypothesis of schizophrenia is due to either the inadequacy or excess of neurotransmitters, which include dopamine, glutamate, and serotonin [1]. Generally, the receptor antagonists of the same are used as anti-schizophrenic or antipsychotic drugs, which are classified into the first, second, and third generations. First-generation drugs act only on dopaminergic receptors causing extrapyramidal side effects (EPS), whereas second-generation drugs (atypical) antipsychotic drugs act on 5-HT_2A_ receptors and dopaminergic receptors, resulting in little EPS. Second-generation drugs have become the first-line treatment for schizophrenia. Among second-generation drugs, zotepine (ZTP) is one of the most effective antipsychotics that acts against dopamine (D2) receptors and 5-HT_2A_ receptors and inhibits norepinephrine reuptake [2,3,4]. ZTP has the advantage of causing reduced EPS than many other antipsychotics. ZTP was introduced in Japan (1982); it was approved in the UK, Germany, and ten other Asia and European countries. However, ZTP is not approved by the US FDA even after reanalysis in 2016 as it has not reached the threshold effect [5]. Nor-zotepine, a major metabolite (30–40%) of ZTP, is therapeutically active but 4–5 times less potent than its parent molecule. ZTP (molecular weight: 331.86 g/mol) is a BCS class II drug with low water solubility (0.046 µg/L) and high *log P* (4.8) is marketed as Sirilept, Zotipax, and Lodopin tablets. Therapeutic use of ZTP has been limited due to lower blood–brain barrier (BBB) permeability and P-glycoprotein (P-gp)-mediated efflux [6]. Owing to its low rate of dissolution with substantial first-pass metabolism (70%), the oral bioavailability is limited (7–13%) [7]. 

The central nervous system (CNS) poses a significant hurdle for oral delivery in schizophrenia due to the blood–brain barrier and P-gp efflux transporters. In such circumstances, a higher dose is required to obtain therapeutic concentrations, which leads to increased adverse effects. Though oral drug delivery is superior concerning patient compliance, limitations to the slow onset of action, low bioavailability, pre-systemic metabolism, absorption barriers, and rapid clearance make it inefficient for brain targeting. When ZTP is administered orally, there are two significant problems. Firstly, the brain restricts any foreign substance from entering it due to the complexity of the BBB and the high expression of efflux transporters in the endothelial layer [8,9]. Second, the drug is available in a tablet at doses ranging from 25–100 mg/day with a maximal daily dose of 300 mg/day. Hence, to improve the bioavailability, safety, and efficacy of delivery systems alternative routes of administration should be preferred. Intranasal (IN) delivery is a potential administration route for targeting drugs to both systemic and CNS, avoiding first-pass metabolism [10,11]. Although ZTP has dose-associated adverse effects, it is more effective than other antipsychotics [12]. By intranasal route of administration, the dose of ZTP can be reduced yet achieve the therapeutic concentration at the site of action with reduced side effects. Novel drug delivery strategies including lipid nanoparticles, polymeric nanoparticles, dendrimers, and nanocrystals have been explored to transport drugs to the brain. Among the various novel delivery systems, the microemulsion is considered a better strategy owing to its enhanced drug solubility and bioavailability [13,14,15]. A few researchers conducted research to overcome the drawbacks of oral delivery of ZTP as cyclodextrin complexes and SMEDDS to improve the solubility. However, the authors have not investigated the brain distribution and pharmacodynamics of the formulation [16,17]. 

Our previous research on ZTP nanosuspension intended for intranasal delivery was reported with improved brain distribution [18]. Recently, ZTP-loaded lipid nanoparticles were formulated for oral delivery by Nagaraj et al. [19]. Herein, we propose a microemulsion (ME) as a suitable approach for intranasal drug administration. The ME formulation does not involve using any sophisticated equipment such as a microfluidizer, ball mill, or spray drier. The ability to solubilize a high amount of drug, longer shelf-life, ease of preparation, and high permeability make microemulsion a distinctive formulation approach for ZTP transport via the intranasal route. To the best of our knowledge, till now, there are no MEs that have been reported for brain distribution of ZTP. 

Hence, in this attempt, ZTP-ME was developed and administered via the intranasal route that might improve brain distribution. The objective of the present investigation was aimed to optimize ZTP-ME by the quality by design (QbD) approach. Further, optimized ME was characterized for its physicochemical properties, ex vivo permeation, in vivo pharmacokinetics, and anti-schizophrenic activity studies.

## 2. Materials and Methods

### 2.1. Materials

Zotepine was a gift sample from Symed Laboratories, Hyderabad, India. Capmul MCM C8 was a kind gift sample from Abitech Pharma, Mumbai, India. Capryol PGMC, Labrasol, Labrafil 1994, Labrafac, and Transcutol HP were acquired from Gattefose India Pvt. Ltd., Mumbai. Tween-20, Tween-80, oleic acid (OA), isopropyl myristate (IPM), polyethylene glycol (PEG)-400, ammonium acetate, calcium chloride (CaCl_2_), sodium chloride (NaCl), and potassium chloride (KCl) were purchased from TCI Chemicals Pvt. Ltd., Hyderabad, India. Eagle’s Minimum Essential Medium (EMEM), Dulbecco’s Modified Eagle’s Medium (DMEM), and non-essential amino acids and trypsin-EDTA solution were procured from Sigma-Aldrich, St. Louis, MO, USA. All the solvents used for analysis are HPLC grade from Merck^®^, Mumbai, India

### 2.2. Solubility of ZTP in Various Vehicles 

The solubility of ZTP was studied in various oils, surfactants, and co-solvents by adding an excess amount of the drug to the respective vehicle. The samples were kept in a shaker incubator (100 rpm) at 37 °C for 48 h, followed by centrifugation at 12,000 rpm for 10 min. The supernatant was collected and diluted appropriately with methanol and analyzed using the HPLC technique [20]. 

### 2.3. Formulation Development

#### 2.3.1. Construction of Pseudo Ternary Phase Diagram

The pseudo-ternary phase diagram was constructed with oil and S_mix_ (surfactant: co-surfactant) to identify the mono-phasic region of the microemulsion [20]. The highest ZTP solubility showing oil and S_mix_ was chosen for preparing ME. S_mix_ was prepared in different weight ratios of surfactant: co-surfactant (1:1, 2:1, and 3:1% *w/w*). Subsequently, the combination of oil and S_mix_ varied from 1:9 to 9:1 (% *w/w*). Each mixture was diluted with distilled water drop-wise under a vortex, and dilution was discontinued when turbidity or gel formation was observed [20,21]. The mixtures were observed visually to determine the formation of crude emulsion or gel consistency. Pseudo-ternary diagrams were constructed with Pro Sim Ternary software. The S_mix_ ratio, which displayed the maximum monophasic emulsification region, was considered for preparing ME.

#### 2.3.2. Optimization of ZTP Loaded ME by Quality by Design (QbD) Approach

Although the pseudo-ternary diagram gives an insight into the monophasic emulsion region of the prepared ME, the composition that gives the desired droplet size and PDI with the least possible surfactant concentration can be identified with the use of Design-Expert statistical software. The characteristics of ME are dependent on the relative amounts of each excipient, provided the final volume of the formulation is unchanged. Hence, the I-optimal mixture model (Design-Expert^®^ software, version 11.0.2.0) was utilized in this study. The selected design provides an advantage of the model’s minimized average prediction variance [22]. The model was composed of independent variables: X_1_: oil, X_2_: S_mix_, and X_3_: water. Each factor was used at upper and lower levels on the ternary-phase diagram. The responses globule size (Y_1_) and PDI (Y_2_) were set to determine the effect of variables [23]. The data obtained were subjected to various quadratic, linear, special quadratic, and special cubic models. Based on the regression coefficient, the best-fitting model was selected. ANOVA was applied to determine the significant factor affecting the responses. Further, optimization was performed by numerical and graphical methods [24]. Validation of the optimization study with three confirmatory runs was performed for a high degree of prognostic ability of design and the outcomes acquired were compared with that of expected values [25].

#### 2.3.3. Preparation of ZTP-ME

A series of ZTP-ME formulations were prepared by the water-titration method as per the compositions given in Table 1. In brief, 20 mg of ZTP was solubilized in each mixture of oil and S_mix_ on a vortex mixer and titrated with deionized water [26]. The prepared ME was stored for further characterization.

### 2.4. Physicochemical Characterization of ZTP-ME

#### 2.4.1. Determination of Globule Size, PDI, and Zeta Potential (ZP)

The mean globule size, PDI, and ZP of ZTP-ME were investigated by a zeta sizer (Zetasizer Nano ZS 90, Malvern Instruments Ltd., Worcestershire, UK). All the samples were analyzed after 100 times diluted with water at 25 °C [27]. 

#### 2.4.2. Drug Content and pH

The optimized ZTP-ME formulation was dispersed in methanol using a magnetic stirrer at 500 rpm for 24 h. The resulted solution was centrifuged, and the supernatant was collected. Further, drug content was analyzed at λmax of 264 nm using the HPLC method (see details in Supplementary Method, S1). The pH of the optimized ZTP-ME was measured using a digital pH meter (Mettler Toledo).

Drug content=[Amount of ZTP in ME/Total weight of ME]×100

#### 2.4.3. Freeze-Thaw Stability and Long-Term Stability Studies

The optimized formulation was subjected to a total of three complete freeze–thaw cycles. Each cycle consists of 12 h at 40 °C and is followed by 12 h at −20 °C. The globule size and PDI were analyzed before and after the study [28]. The optimized ZTP-ME was stored at 30 ± 2 °C/65% RH for 6 months as per ICH guidelines. At regular time intervals, the samples were checked by visual observation for any phase separation, creaming, or flocculation. Finally, globule size, PDI, ZP, and drug content were analyzed [29].

### 2.5. Ex Vivo Studies

#### 2.5.1. Ex Vivo Permeation

The porcine nasal mucosa without septum was freshly excised and collected from a local slaughterhouse. The superior nasal membrane was detached from the nasal cavity and then adhered tissues were separated and immediately stored in ice-cold PBS pH 6.4 with aeration. [30]. Before experimentation, the mucus membrane was soaked in simulated nasal fluid pH 5.5 to saturate for 30 min and to wash off any soluble components adhering to the membrane [28]. The experiment was conducted with the Franz diffusion cell (Orchid, EMFDC08, Nashik, India). The receptor chamber remained filled with SNF (28 mL) as a permeation medium. The excised nasal mucosal membrane (1 mm thick) was mounted in between the two compartments, and test samples (ZTP-ME, and ZTP solution in ethanol and propylene glycol (1:4)) were added to the donor compartment. Later, 2 mL of samples was collected at prefixed time points (0.25, 0.5, 0.75, 1.0, 1.5, 2.0, 3.0 and 4.0 h) and fresh medium was added after every time point. All the samples were analyzed using the HPLC method [26,28]. The percentage of drug diffused against the time graph was plotted. The formula used to calculate the permeability coefficient is as follows:

$$P=\frac{\mathrm{dQ}}{\mathrm{dt}}\times C_{o}A$$

where dQ/dt is the permeability rate (mg/h); C_o_ is the initial concentration; A is the sufficient surface area for permeation.

#### 2.5.2. Nasal-Cilia Toxicity by Histopathology

Freshly extracted porcine nasal mucosa was placed in SNF (pH 5.5). The nasal mucosal membrane of 1.5 cm^2^ diameter with an equal thickness (1 mm) was labeled as a negative control (SNF), positive control (isopropanol), plain drug, and ZTP-ME, respectively. Each membrane was placed on the diffusion cell receptor compartment and treated with the respective samples (Eq. 1.5 mg ZTP). After 4 h, the treated mucosal membranes were flushed with PBS and fixed in buffered formalin for histopathology studies to evaluate the toxicity [26,31,32]. 

### 2.6. In Vitro Cytotoxicity

A cytotoxicity test was performed on the human nasal epithelial cell line, RPMI 2650 (NCCS, Pune, India) [33]; human bronchus cell line, Beas-2B (ATCC, University Boulevard Manassas VA, USA) [34]; and mouse neuroblastoma cell line, Neuro-2A (ATCC) [35]. RPMI 2650 and Neuro-2A cells were nurtured in EMEM and added with 10% fetal bovine serum. Beas-2B cells were cultured in a 1:1 ratio of low glucose DMEM and Ham’s F-12K medium with 10% fetal bovine serum. The cells were stabilized with 1% non-essential amino acids at 5% CO_2_/37 °C/90% relative humidity in the incubator. Cells were treated with 0.25% trypsin/1 mM EDTA solution after reaching 80–90% of confluence.

The cytotoxic evaluation of the ZTP, ZTP-ME, and blank ME was performed. RPMI 2650, Beas-2B, and Neuro-2A cell lines were subjected to MTT assay to determine the cell viability. The cells were treated with ZTP (in DMSO) and ZTP-ME at a concentration range of 7.25–500 µg/mL, and blank ME was equivalent to the volume of ZTP-ME. The cells were incubated for 48 h before adding 100 µL of MTT (0.5 mg/mL) to each well. The cells were incubated at 37 °C for 4 h, after which they formed formazan crystals. The clear liquid was discarded from all the wells; formazan crystals were liquified in DMSO, and the absorbance was noted at 570 nm. The ratio of cell viability was calculated, and the concentration of test drug impeding cell growth by 50% (IC_50_) was estimated from dose–response curves using Graph Pad Prism software (version 6.0).

### 2.7. In Vivo Pharmacokinetic Study

#### 2.7.1. Animal Experimentation

Male Wistar rats weighing around 230–260 g were employed for pharmacokinetics and were maintained in the light-controlled area at 22 ± 2 °C with a standard diet and water. The study protocol (NIP/01/2018/PE/263) was authorized by CPCSEA and IAEC for the Care and Use of Laboratory Animals [18]. Rats were accustomed to an in-house animal room for one week before experimentation and separated into four groups at random (*n* = 8). Group A animals were given intravenous (IV) zotepine solution (1:4 ethanol and propylene glycol); group B animals were dosed with pure zotepine drug via the intranasal (IN) route; group C received intranasal microemulsion (IN ME); and group D received oral ME [36,37]. All the animals were administered with ZTP at a dose corresponding to 4.4 mg/kg. The animals receiving IN were anesthetized with isoflurane before administration into the nostrils using a micropipette (10–100 µL) by holding the animal in an inclined posture [38]. 

#### 2.7.2. Sample Collection, and Processing

Blood samples were collected under mild anesthesia, and brain samples were removed immediately from the euthanized (CO_2_ exposure) animal at pre-determined time intervals (0.5, 1, 2, 4, 6, 8, 12, 24 h). Plasma was isolated from blood samples through centrifugation at 8000 rpm for 10 min/4 °C and maintained at −20 °C until further evaluation. The collected brain tissues were cleaned with PBS (pH 7.4) to clear out fluid, if any, blotted up using a filter paper, and kept at −80 °C for further evaluation. 

ZTP was extracted from plasma samples by protein precipitation method in which acetonitrile was added to plasma in a 4:1 ratio, and the drug was extracted into acetonitrile. The samples were mixed by vortex mixer followed by centrifugation at 12,000 rpm for 10 min/10 °C to separate the clear liquid for analysis. Brain samples were homogenized with 1.5 mL of PBS:ACN (1:1) by employing a Teflon homogenizer (Remi Motor, India) and centrifugation at 12,000 rpm for 15 min/10 °C [28,39,40]. The clear liquid was separated; an internal standard was added and parched employing a nitrogen (inert gas) evaporator (Biotage, TurboVap^®^LV). The dried specimens were re-formed with acetonitrile (250 μL) followed by centrifugation, and the transparent liquid was spiked (100 μL) into a chromatographic instrument for analysis [28]. 

#### 2.7.3. Bioanalytical Method Development and Validation

The HPLC method was established to investigate bio-samples using the HPLC system (Waters Corporation, Leederville, WA, USA) with a photodiode array detector (PDA). The column employed for the investigation was the Fortis column (C18). The mobile phase employed was ammonium acetate (20 mM):acetonitrile:methanol in the proportions: 10:45:45. The flow rate was adjusted at 1 mL/min. The wavelength used was 264 nm. From the stock solution (1 mg/mL), subsequent dilutions of ZTP were prepared and analyzed. Validation was conducted using parameters established in the field (linearity, accuracy, precision, LOD, and LOQ). Recovery was deliberated using ZTP in the blank plasma or brain homogenate at 0.1, 0.5, 1, 2, 4, 6, 8, and 10 μg/mL concentrations. Analysis of samples was performed, and a comparison was made between extracted and unextracted concentrations [21]. A standard curve was delineated, plotting concentration on the x-axis and a ratio of the area obtained on the y-axis. 

#### 2.7.4. Pharmacokinetic Parameters

Pharmacokinetic parameters such as AUC_0–t24_; highest concentration achieved, C_max_; time to attain the maximal concentration, T_max_; mean residence time, MRT; and half-life, t_1/2_ were analyzed by Phoenix Win Nonlin software (version 8.1). The following parameters were estimated to assess the brain targeting efficiency of the optimized drug-loaded ME [41].

$$(i)\;\mathrm{Drug}\;\mathrm{targeting}\;\mathrm{efficiency}\;\left(\%\mathrm{DTE}\right)=\left(B_{\mathrm{IN}}/P_{\mathrm{IN}}\right)/\left(B_{\mathrm{IV}}/P_{\mathrm{IV}}\right)\times 100$$

$$(\mathrm{ii})\;\mathrm{Drug}\;\mathrm{transport}\;\mathrm{percentage}\;\left(\mathrm{DTP}\right)=\left(\frac{\left(B_{\mathrm{IN}}-B_{x}\right)}{\left(B_{\mathrm{IN}}\right)}\right)\times 100$$

B_x_ = B_IV_/P_IV_ × 100

B_IV_ = area under curve_0–t_ in the brain resulted from intravenous administration;
P_IV_ = area under curve_0–t_ in the blood resulted from intravenous administration;
B_IN_ = area under curve_0–t_ in the brain resulted from intranasal administration;
P_IN_ = area under curve_0–t_ in the blood resulted from intranasal administration.

### 2.8. In Vivo Anti-Schizophrenic by Catalepsy Test

The catalepsy test has been used as a predictive tool to measure extrapyramidal side (EPS) effects in animals treated with anti-schizophrenic drugs. It is a behavioral disorder distinguished by its rigidity [42,43]. The cataleptic activity was assessed at pre-determined time points (5, 15, 30, 45, 60, 90, 120, and 240 min) by the block test after intranasal and oral administration of ZTP-ME (4.4 mg/kg) in rats. In brief, for the block test, the forelimbs of rats were placed on a 10 cm wooden platform, and the time taken for retraction of forelimbs was measured. The retraction time was recorded for three consecutive attempts at each time point. Scoring was given from 1–6; 6 scores for maximum intensity response based on the time taken for retraction (1 = 0–10 s, 2 = 11–20 s, 3 = 21–30 s, 4 = 31–40 s, 5 = 41–50 s, and 6 = 50–180 s) and a scoring of ≥3 was considered cataleptic [44,45]. 

### 2.9. Statistical Analysis 

The entire data are represented as the mean ± S.D. The outcome of the data was illuminated by Prism software (version 9.0; Graph Pad, San Diego, CA, USA) by treating the data to two-way ANOVA succeeded by Tukey’s–Kramer and Bonferroni’s multiple correlation test. The result was regarded as significant at *p* < 0.05.

## 3. Results and Discussion

### 3.1. Solubility of ZTP in Various Vehicles 

**Figure 1:**
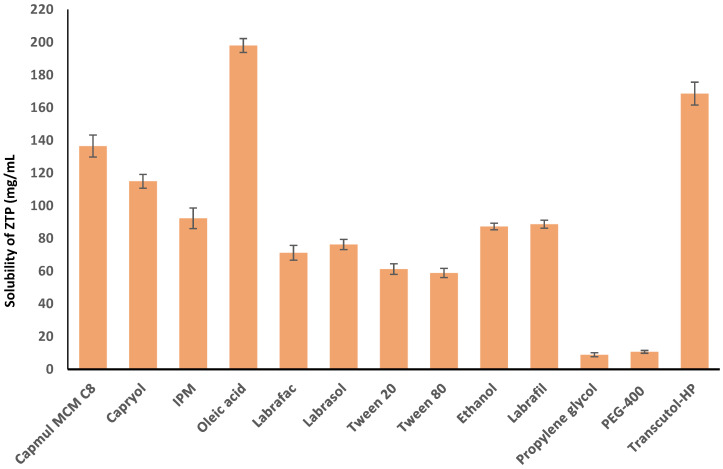
Solubility of ZTP in various vehicles (mean ± SD, *n* = 3).

The drug solubility in the oil phase is vital for the maximum loading of ZTP in the microemulsion system. The solubility of ZTP among various oils was seen in the decreasing order of Oleic acid > Capmul MCM C8 > Capryol PGMC > IPM, as shown in Figure 1. ZTP has a solubility of 198 ± 4.23 mg/g and 136.5 ± 6.72 mg/g in Oleic acid and Capmul MCM C8, respectively. The high solubility in Oleic acid could be attributed to the hydrophobic interaction and ionic interaction between ZTP and Oleic acid [46]. The solubility of ZTP might be high in Capmul MCM C8 (glyceryl monocaprylate) due to high caprylic content [47]. Hence, both oils were selected for constructing pseudo-ternary diagrams in formulating ZTP-ME.

The selection of surfactants is crucial as it aids in lowering interfacial tension by creating a layer at the interface of oil and water, which results in the impetuous generation of a microemulsion [48]. Surfactants with an HLB value of 10 and higher are essential in forming stable ME. Besides, the surfactant should have a higher solubilization capacity for the selected drug. Among the surfactants tested, ZTP was highly soluble (76.3 ± 3.14 mg/g) in Labrasol (HLB = 14). Labrasol was also reported to augment the permeability and bioavailability of the drugs [20,49]. However, surfactant alone may not be sufficient to attain a required transient negative interfacial tension [50]. Hence, a co-surfactant was used to decrease the interfacial tension to form a stable ME with improved flexibility and curvature of globules. The solubility of ZTP was high (168.58 ± 7.01 mg/g) in Transcutol-HP (HLB 4) in comparison with PEG-400 (10.7 ± 0.84 mg/g). Thus, Transcutol-HP was selected as a co-surfactant for developing microemulsions.

### 3.2. Formulation Development

#### Pseudo Ternary Phase Diagram

**Figure 2:**
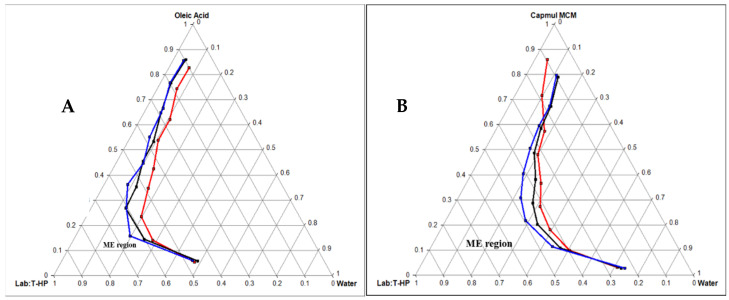
Pseudo-ternary phase diagram of Oleic acid oil (**A**) and Capmul MCM EP oil (**B**) with S_mix_ ratio (Labrasol and Transcutol-HP = 1:1 in red line, 2:1 in black line, and 3:1 in blue line).

Based on the above solubility study data, both Oleic acid and Capmul MCM C8 were selected as oil. Labrador and Transcutol HP were selected as a surfactant and co-surfactant, respectively. Pseudo-ternary diagrams were created with Oleic acid and Capmul MCM C8 as oil phases individually while keeping S_mix_ (Labrasol:Transcutol HP) the same. The oil and S_mix_ were mixed in weight ratios (1:9, 2:8, 3:7, 4:6, 5:5, 6:4, 7:3, 8:2, and 9:1). Figure 2A,B indicates the phase diagrams of Oleic acid and Capmul MCM C8, respectively. The ternary diagram plotted with Labrasol/Transcutol-HP as S_mix_ (1:1) unveiled a maximum emulsion region with Capmul MCM C8. Hence, the same was chosen for the optimization of ME. 

### 3.3. Optimization of ZTP-ME by QbD

Mixture designs belonging to a particular type of response surface optimization with the minimum number of runs were chosen. These designs are primarily used in industries since most product characteristics are a function of excipients’ composition. As per the I-optimal design, 12 runs were the recommended design; three factors, the % *w/w* ratios of oil:S_mix_:water from 10:40:10 (low/−1) to 50:60:40 (high/+1) were considered to obtain desired responses of particle size and PDI. 

### 3.4. Physicochemical Characterization of ZTP-ME

#### 3.4.1. Globule Size, PDI, and Zeta Potential (ZP) 

The globule size of the formulations varied from 90.1 ± 3.43 to 238.3 ± 5.65 nm (Table 1), and the fit summary suggested the linear model. The model was noteworthy with an inconsequential lack of fit. An F value of 46.66 implies the 0.01% probability of noise affected the results. The consequence of the response factors was assessed by ANOVA, where independent variables with a *p*-value smaller than 0.05 were deliberated to be notable. In this case, factors X_1_: oil and X_3_: water had a significant impact on globule size, and the obtained regression equation was globule size = 4.97 X_1_ − 0.82 X_2_ + 2.82 X_3_.

**Figure 3:**
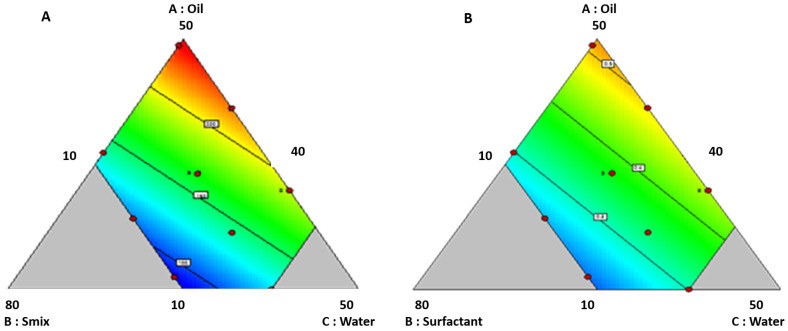
(**A**) Contour plots displaying the effect of variables on size; (**B**) contour plots displaying the effect of variables PDI.

As shown in the contour plot in Figure 3A, globule size increases with increased oil content. This increase might be due to the expansion of an oil drop. Factor X_3_ (water) plays a significant role in controlling the globule size. An increase in globule size with high water content was observed. This increase might be due to a reduction in the interfacial tension with the rise in water content. At the same time, globule size has reduced with a surge in S_mix_ concentration, which might be due to decreased interfacial tension. Further R^2^, adjusted R^2^, and predicted R^2^ of the model were 0.912, 0.892, and 0.841. Furthermore, a precision value of 19.52 indicates the model’s capability for further optimization. The effect of the selected variables on the globule size is in accordance with previous studies [51].

PDI of the developed formulations varied from 0.171 ± 0.023 to 0.584 ± 0.011; the fit summary suggested the linear model. The model was notable with an inconsequential lack of fit, with an F value of 7.21, implying the 1.35% probability of noise affected the results. ANOVA evaluated each factor’s significance on the response. Factors X_1_: oil and X_3_: water significantly impacted PDI. The regression equation is as follows: PDI = 0.010 X_1_ − 0.001 X_2_ + 0.007 X_3_

As shown in the contour plot in Figure 3B, PDI increases with increasing the oil and water content. The main reason might be compromising the concentration of the Smix efficiency in forming a stable homogenous system. For numerical optimization, globule size and PDI criteria were set as not more than 150 nm and 0.312, respectively. The desirability of 0.720 was obtained for a composition (*w/w*) of oil, S_mix_, and distilled water in 10%, 50%, and 40%. A globule size of 124 nm and a PDI of 0.312 was achieved for this composition. Further, graphical optimization was performed, and the design space was obtained. Formulations with a zeta potential of more than + 30 mV or more than −30 mV are generally considered stable colloidal formulations [52]. The ZP of all the prepared ME was in the range of 35 to 43 mV. This value is a consequence of drug loading, indicating that all the formulations are stable. As there is no significant difference in ZP among various formulations, the same was not considered in the design.

#### 3.4.2. pH and Drug Content

The pH of all the formulations was observed to be in the range of 5.9–6.2, which is a normal pH range of the nasal cavity (pH 5.5–6.5). A significant difference in nasal pH will irritate the nasal mucosa. Hence, formulation pH should be maintained in the optimal range [53]. The drug content in all the formulations was >98%, indicating a high loading capacity of ME. 

#### 3.4.3. Freeze-Thaw Stability and Long-Term Stability Studies 

A freeze–thaw stability test was conducted to determine the influence of temperature on the phase stability of ZTP-ME, as a change in equilibrium with low and high temperatures can cause instability [54]. No physical changes (turbidity, phase separation, or drug precipitation) were observed following the freeze–thaw study, indicating the thermostability of ZTP-ME. Moreover, freeze–thaw conditions did not affect globule size (131.48 ± 2.29 nm), PDI (0.22 ± 0.05), ZP (33.13 ± 1.37 mV), and drug content (97.7 ± 0.6%) of optimized ZTP-ME. 

The stability data of the optimized ZTP-ME is represented in Table 2. The formulation was found to be stable over 6 months with respect to particle size, PDI, ZP, and drug content ranging from 124.6 ± 7.33 nm to 120.3 ± 3.48 nm, 0.31 ± 0.02 to 0.17 ± 0.03, 38.7 ± 3.01 to 33.5 ± 3.28 mV, 98.92 ± 0.74% to 97.73 ± 0.63%, respectively. ZTP-ME was stable up to 12 months without any considerable variation in particle size and drug content. However, a decrease in PDI was observed during long-term stability studies, which might be due to the stabilizer’s inability to maintain the system’s homogeneity for such a long period. 

### 3.5. Ex Vivo Studies

#### 3.5.1. Ex Vivo Permeation

**Figure 4:**
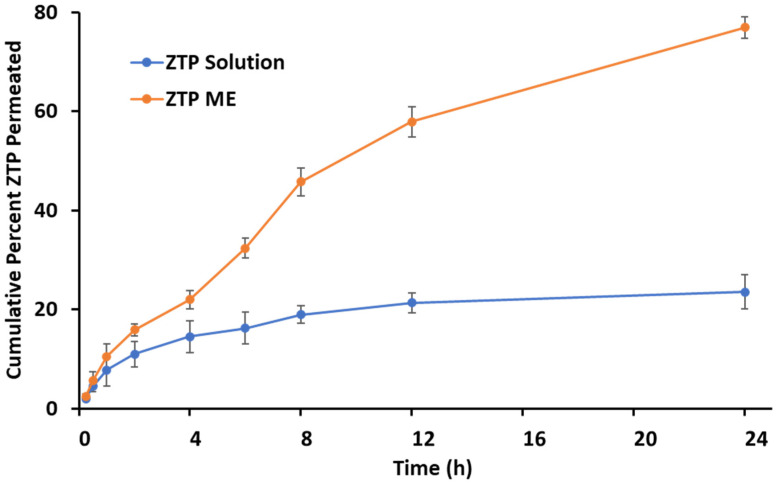
Ex vivo permeation of ZTP-ME and ZTP solution across porcine nasal mucosa (mean ± SD, *n* = 3).

The ex vivo permeation study was accomplished with the porcine nasal mucosa and the graphical representation as shown in Figure 4. Diffusion coefficients were calculated for the ZTP solution and ZTP-ME. The ZTP solution revealed a lower permeability coefficient ((2.476 ± 0.13) × 10^−5^ cm^2^/min) compared to the ZTP-ME ((6.650 ± 0.27) × 10^−5^ cm^2^/min) which confirms the enhanced permeability of ME (*p* < 0.01). The increase in permeability was attributed to the use of a combination of vehicles in ME, including Capmul MCM C8 [55,56], Labrasol, and Transcutol-HP [57,58]. 

#### 3.5.2. Nasal-Cilia Toxicity by Histopathology

**Figure 5:**
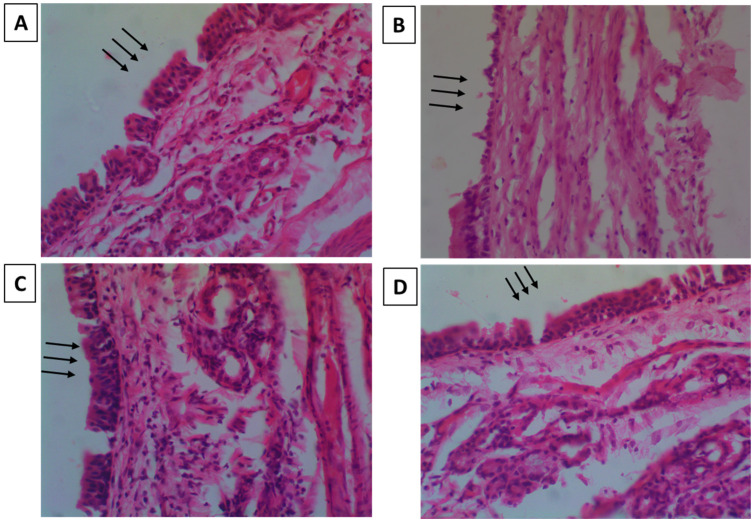
Microscopic images showing nasal-cilia toxicity of (**A**) negative control (arrows indicating intact mucosa); (**B**) positive control (arrows indicating damaged mucosa); (**C**) ZTP solution (arrows indicating intact mucosa); and (**D**) ZTP-ME (arrows indicating intact mucosa).

Nasal-cilia toxicity was checked on rats to examine the impact of ZTP-ME on the nasal mucosa’s durability. Figure 5A–D shows the histology of the negative control, positive control, plain drug, and ZTP-ME-treated mucosa, respectively. Nasal mucosa treated with isopropanol exhibited massive damage with changes on the epithelial surface and internal tissue damage. Mucosa treated with SNF was observed to be undamaged with a conserved structure. Mucosa treated with ZTP-ME showed neither cell necrosis nor physical impairment, which indicates that the ZTP formulations showed no harmful effects on the nasal mucosa. 

### 3.6. In Vitro Cytotoxicity 

We have investigated the safety of PD, BME, and ZTP-ME on the human nasal cell line, RPMI 2650; human bronchus cell line, Beas-2B, and mouse neuroblastoma cell line, Neuro-2A as in vitro models. The IC_50_ values of the plain drug and formulations on various cell lines are given in Table 3. As the mucosal layer in the nasal cavity is sensitive, a toxicity test was performed on RPMI 2650 to assess the formulation’s safety. When the drug is administered via the intranasal route, some amount of the drug may reach the lungs. Hence, a cytotoxicity test was performed on human bronchus cell lines. The drug reaches the brain in higher concentrations when administered intranasally, leading to neuronal cell death. RPMI 2650 cells were used as a primary screening means for cytotoxicity and penetrability in preclinical assessment for intranasal drug delivery [33].

**Figure 6:**
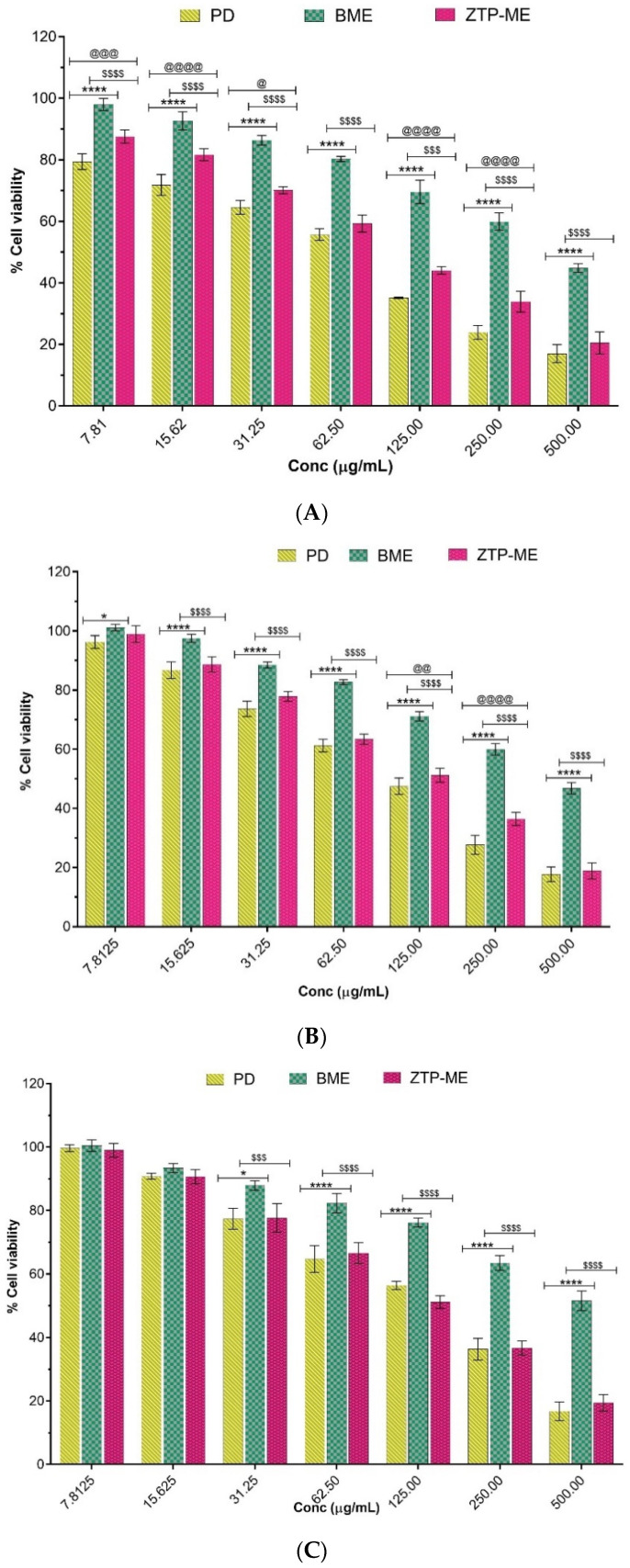
(**A**) Cytotoxicity of PD, BME, and ZTP-ME on RPMI 2650 cell lines (7.81–500 µg/mL) for 48 h followed by MTT incubation. The values presented were the mean ± S.E.M (*n* = 3). Abbreviations: PD = plain drug, BME = blank microemulsion, ZTP-ME = zotepine microemulsion. **** *p* < 0.0001 PD vs. BME, ^@@@^ *p* < 0.001 PD vs. ZTP-ME, ^$$$$^ *p* < 0.0001 BME vs. ZTP-ME (7.81 µg/mL);**** *p* < 0.0001 PD vs. BME, ^@@@@^ *p* < 0.0001 PD vs. ZTP-ME, ^$$$$^ *p* < 0.0001 BME vs. ZTP-ME (15.62 µg/mL); **** *p* < 0.0001 PD vs. BME, ^@^ *p* < 0.01 PD vs. ZTP-ME, ^$$$$^ *p* < 0.0001 BME vs. ZTP-ME (31.25 µg/mL); **** *p* < 0.0001 PD vs. BME, *p* > 0.05 PD vs. ZTP-ME, ^$$$$^ *p* < 0.0001 BME vs. ZTP-ME (62.5 µg/mL); **** *p* < 0.0001 PD vs. BME, ^@@@@^*p* < 0.0001 PD vs. ZTP-ME, ^$$$^ *p* < 0.001 BME vs. ZTP-ME (125 µg/mL); **** *p* < 0.0001 PD vs. BME, ^@@@@^ *p* < 0.0001 PD vs. ZTP-ME, ^$$$$^ *p* < 0.0001 BME vs. ZTP-ME (250 µg/mL); **** *p* < 0.0001 PD vs. BME, ^$$$$^ *p* < 0.0001 BME vs. ZTP-ME (500 µg/mL). (**B**) Cytotoxicity of PD, BME, and ZTP-ME on Beas-2B cell lines (7.81–500 µg/mL) for 48 h followed by MTT incubation. The values presented were the mean ± S.E.M (*n* = 3). Abbreviations: PD = plain drug, BME = blank microemulsion, ZTP-ME = zotepine microemulsion. * p < 0.05 PD vs. BME, *p* > 0.05 PD vs. ZTP-ME, *p* > 0.05 BME vs. ZTP-ME (7.81 µg/mL); **** *p* < 0.0001 PD vs. BME, *p* > 0.05 PD vs. ZTP-ME, ^$$$$^ *p* < 0.0001 BME vs. ZTP-ME (15.62 µg/mL); **** *p* < 0.0001 PD vs. BME, *p* > 0.05 PD vs. ZTP-ME, ^$$$$^ *p* < 0.0001 BME vs. ZTP-ME (31.25 µg/mL); **** *p* < 0.0001 PD vs. BME, *p* > 0.05 PD vs. ZTP-ME, ^$$$$^ *p* < 0.0001 BME. vs. ZTP-ME (62.5 µg/mL); **** *p* < 0.0001 PD vs. BME, ^@@^ *p* < 0.01 PD vs. ZTP-ME, ^$$$$^ *p* < 0.0001 BME vs. ZTP-ME (125 µg/mL); **** *p* < 0.0001 PD vs. BME, ^@@@@^ *p* < 0.0001 PD vs. ZTP-ME, ^$$$$^ *p* < 0.0001 BME vs. ZTP-ME (250 µg/mL); **** *p* < 0.0001 PD vs. BME, ^$$$$^ *p* < 0.0001 BME vs. ZTP-ME (500 µg/mL). (**C**) Cytotoxicity of PD, BME, and ZTP-ME on Neuro-2A cell lines (7.81–500 µg/mL) for 48 h followed by MTT incubation. The values presented were the mean ± S.E.M (*n* = 3). Abbreviations: PD = plain drug, BME = blank microemulsion, ZTP-ME = zotepine microemulsion. *p* > 0.05 PD vs. BME, *p* > 0.05 PD vs. ZTP-ME, *p* > 0.05 BME vs. ZTP-ME (7.81 µg/mL); *p* > 0.05 PD vs. BME, *p* > 0.05 PD vs. ZTP-ME, *p* > 0.05 BME vs. ZTP-ME (15.62 µg/mL); * *p* < 0.01 PD vs. BME, *p* > 0.05 PD vs. ZTP-ME, ^$$$^ *p* < 0.001 BME vs. ZTP-ME (31.25 µg/mL); **** *p* < 0.0001 PD vs. BME, *p* > 0.05 PD vs. ZTP-ME, ^$$$$^ *p* < 0.0001 BME vs. ZTP-ME (62.5 µg/mL); **** *p* < 0.0001 PD vs. BME, ^$$$$^ *p* < 0.0001 BME vs. ZTP-ME (125 µg/mL); **** *p* < 0.0001 PD vs. BME, ^$$$$^ *p* < 0.0001 BME vs. ZTP-ME (250 µg/mL); **** *p* < 0.0001 PD vs. BME, ^$$$$^ *p* < 0.0001 BME vs. ZTP-ME (500 µg/mL).

Beas-2B and Neuro-2A cell lines were used to study the toxicity of compounds targeting the brain via the nasal route [34,35]. Hence, the cytotoxicity test was conducted on a neuronal cell line to ensure neuronal cells’ safety. The percentage cell viability of individual cells after treatment is shown in Figure 6A–C. 

Cytotoxicity of prepared microemulsions appeared to be dose dependent. BME showed high IC_50_ values in all three cell lines, indicating that the excipients used in the study are safe for preparing drug-loaded ME. Compared with PD, the IC_50_ values of ZTP-ME were not significantly different in the Beas 2B and RPMI 2650 cell lines. However, a significant decrease in IC_50_ values of ZTP-ME compared with PD in Neuro 2A cell lines was attributed to better-enhanced permeation of ZTP in microemulsion form leading to toxicity in the cell lines at low concentration. Though the IC_50_ values of PD and ZTP-ME were significantly low and different from blank formulations, the values were 88–152 µg/mL, far less than the exposure levels in animal humans, when administered via the intranasal route. Thus, it can be confirmed that ZTP-ME does not cause cytotoxicity at these levels of exposure.

### 3.7. In Vivo Pharmacokinetic Study

#### 3.7.1. Bioanalytical Method Development and Validation

A chromatographic separation technique was developed for ZTP in plasma, and the brain homogenates used a mobile phase composed of ammonium acetate (20 mM):acetonitrile:methanol in the ratio of 10:45:45. ZTP and olanzapine (IS) were separated with a retention time of 8.5 ± 0.33 and 4.3 ± 0.25 min, respectively (Supplementary Figure S1). The standard curve’s linearity was noted in 100–10,000 ng/mL concentration order. The limit of detection (LOD) and limit of quantification (LOQ) of the drug in plasma were 50 ng/mL and 75 ng/mL. Whereas in the case of the brain homogenate, the LOD and LOQ were 75 ng/mL and 100 ng/mL. The ZTP and internal standard recovery percentage were >90 for plasma and brain homogenates. 

#### 3.7.2. Pharmacokinetics in Plasma and Brain

**Figure 7:**
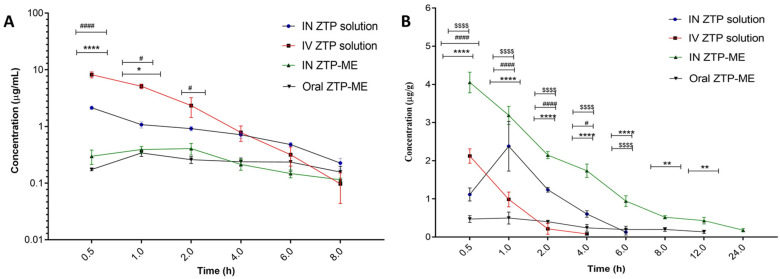
Concentrations of zotepine in (**A**) plasma and (**B**) brain after intranasal and oral administration of ZTP-ME. Data are represented as the mean ± SEM (*n* = 3). **** *p* < 0.0001 IV ZTP solution vs. IN ZTP-ME; #### *p* < 0.0001 IV ZTP solution vs. Oral ZTP-ME^; $$$$^ *p* < 0.0001 IN ZTP-ME vs. Oral ZTP-ME. (*^,#^ *p* ≤ 0.05; ** *p* ≤ 0.01) Abbreviations: IN ZTP—intranasal plain drug, IV ZTP—intravenous plain drug, IN ZTP-ME—intranasal microemulsion, oral ZTP-ME—oral microemulsion.

ZTP concentrations in plasma and brain at different time intervals after administration (IV, IN, and oral) to rats are shown in Figure 7A,B, correspondingly, while its pharmacokinetic parameters are mentioned in Table 4. It was detected that the ME of ZTP has significantly elevated C_max_ and AUC_0–24_ with low T_max_ than both the IV and IN solution of ZTP in the case of concentrations in the brain. As a result, the DTE and DTP percent in ME are larger than in the ZTP pure nasal solution. A higher AUC_0–24_ of ZTP in plasma than in the brain after IV injection reveals that perhaps the drug is widely distributed in plasma, whereas lower doses can migrate to the brain by passive diffusion over a longer period. In this case, it is worth noting that the ME offered a distinct advantage over the ZTP solution in achieving higher concentrations in the brain.

When administered as nasal sprays, drugs are expected to overcome first-pass metabolism. However, our study has not shown such a characteristic increase in plasma concentration of ZTP-ME via the nasal route. The same is in accord with the previous report by Noda K et al. regarding species-related variation in zotepine plasma concentration levels [59]. The study reported a low plasma concentration of zotepine in rats, and similar reports with zotepine nanosuspension via the nasal route were reported in our earlier study [18]. Following the relevant work, the drug absorption from the nasal cavity can usually occur by two pathways: (A) the drug directly enters the brain/CSF from the nasal cavity via olfactory neurons; (B) part of the drug straight away moves into the vascular system and outreaches the brain by traversing the BBB. These mechanisms imply that a portion of ZTP reached the brain following the above two pathways bestow intranasal dosing. Because of its smaller globule size, the ZTP-ME formulation has a high DTE percent and DTP percent, indicating that the medication is immediately transferred to the brain via the transcellular route following an intranasal dosage [27]. 

Besides, the excipients employed in ME contribute to the increasing absorption levels of ZTP. In one investigation, Transcutol^®^ HP was used in self-nanoemulsifying drug delivery systems (SNEDDS) that showed P-gp inhibition activity at 0.60 μg/mL [60]. In the present study, the required P-gp inhibitory concentration of Labrasol and Transcutol-HP was used to impede the P-glycoprotein activity [61,62] and thus helped in improving the bioavailability of ZTP. Besides playing the role of co-surfactant, Transcutol-HP is a non-toxic, biocompatible permeation enhancer. 

### 3.8. In Vivo Anti-Schizophrenic by Catalepsy Test 

**Figure 8:**
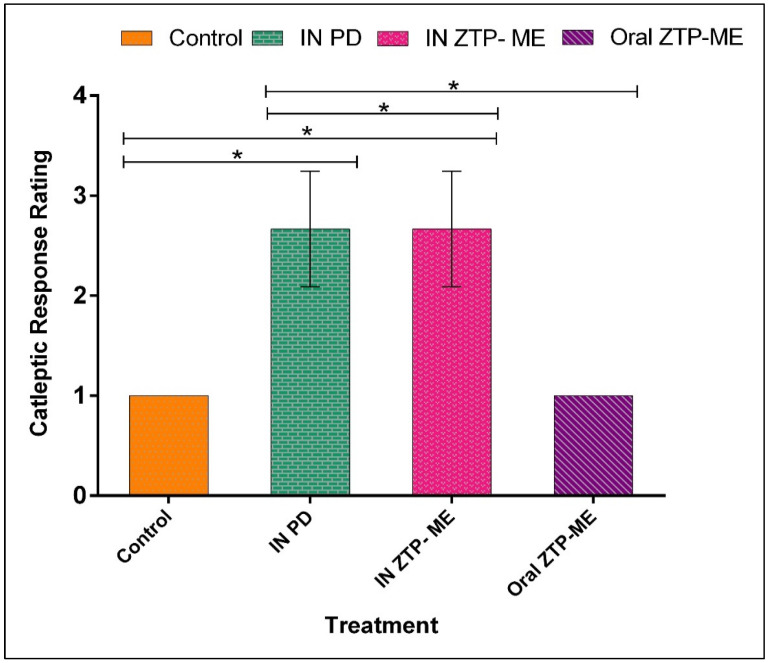
Cataleptic response rating following drug administration based upon the drugs treatment received. The results are expressed as the mean ± standard error (*n* = 3). Cataleptic response score was less than 3 and 2 in intranasal ME and oral ME, hence not considered to be cataleptic (* *p* ≤ 0.05). Abbreviations: IN PD: intranasal plain drug; IN ZTP-ME: intranasal zotepine microemulsion; Oral ZTP-ME: oral zotepine microemulsion.

Catalepsy is described by muscle firmness and fixed position even in the prevalence of external provocations, an extrapyramidal side effect of antipsychotic drugs. The block test results in which rats received ZTP-ME via intranasal and oral routes are given in Figure 8. Animals that received intranasal ME exhibited a higher rigidity in movement followed by oral ME than the control. The reason might be the higher concentrations of ZTP delivered to the brain when administered via the intranasal route. However, both groups were not considered cataleptic as the cataleptic response score was less than 3 and 2 in intranasal ME and oral ME, respectively. The lower score justifies the better efficacy of intranasal ME and suggests a dose reduction to reduce the cataleptic rigor. It was also detected that none of the treated groups suffered from inflammation (hemorrhage of the nasal cavity or neurotoxic effects (e.g., seizures or respiratory system). 

## 4. Conclusions

This study employed the QbD approach and developed ZTP-ME by the water titration method. The intranasal dosing of microemulsion attained significant brain delivery of ZTP. The developed formulation was found to be safe in cell lines and ex vivo tissues. Furthermore, it can be concluded that ZTP-ME administered via the intranasal route is a safe and effective approach to improving brain delivery. The anti-schizophrenic activity test confirms the superiority of the developed formulation via the intranasal route. In comparison to oral delivery, the intranasal route is less complaint; however, considering the bioavailability and therapeutics concentrations in the brain, this approach may prove to be beneficial in attaining the desired pharmacological outcome. 

## Figures and Tables

**Table 1:** Mean globule size, PDI, and zeta potential of ME formulations (mean ± SD, *n* = 3).

Trials	(%*w/w*)	Smix (1:1) (%*w/w*)	Aqueous Phase (%*w/w*)	Response 1 Size (nm)	Response 2 PDI	Response 3 ZP (mV)
1	31.86	58.13	10.22	156.8 ± 3.23	0.282 ± 0.015	37.5 ± 1.52
2	19.09	49.90	31.12	152.9 ±1.23	0.368 ± 0.087	36.2 ± 2.45
3	21.35	60.32	18.64	90.1 ± 3.43	0.171 ± 0.023	40.8 ± 3.35
4	25.82	40.13	34.17	180.5 ± 4.23	0.409 ± 0.015	36.0 ± 4.12
5	38.99	40.22	21.006	238.3 ± 5.65	0.547 ± 0.035	35.1 ± 1.35
6	10.13	50.23	40.12	124.6 ± 3.52	0.212 ± 0.013	38.7 ± 2.35
7	25.82	40.76	34.17	194.3 ± 0.91	0.416 ± 0.077	36.4 ± 3.29
8	12.87	60.24	28.09	92.81 ± 2.81	0.347 ± 0.098	37.3 ± 1.99
9	28.55	49.08	22.35	139.7 ± 1.98	0.216 ± 0.033	40.1 ± 1.66
10	49.22	41.23	10.65	229.2 ± 2.98	0.584 ± 0.011	43.2 ± 4.35
11	28.55	49.08	22.35	163.1 ± 3.54	0.393± 0.017	41.1 ± 3.54
12	28.55	49.08	22.35	173.4 ± 1.34	0.352 ± 0.013	36.6 ± 3.33

**Table 2:** Stability assessment of optimized ZTP-ME for 6 months.

Temperature	Duration	PS (nm)	PDI	ZP (mV)	Drug Content (%)
30 ± 2 °C/65% RH	0 day	124.6 ± 7.33	0.21 ± 0.013	38.7 ± 2.35	98.92 ± 0.74
	15 days	118.5 ± 5.91	0.18 ± 0.032	31.9 ± 2.83	99.42 ± 0.53
	1 month	116.6 ± 5.42	0.15 ± 0.052	29.7 ± 3.62	98.67 ± 0.24
	3 months	121.2 ±9.29	0.19 ± 0.064	36.1 ± 4.18	97.24 ± 0.47
	6 months	120.3 ± 3.48	0.17 ± 0.037	33.5 ± 3.28	97.73 ± 0.63

**Table 3:** IC_50_ values of plain drug and formulations on various cell lines. PD = plain drug, BME = blank microemulsion, ZTP-ME = zotepine microemulsion.

Samples	Neuro 2A	Beas 2B	RPMI 2650
PD	142.46 ± 9.88	88.08 ± 5.29	93.55 ± 13.91
BME	193.93 ± 8.10	155.9 ± 8.91	188.66 ± 7.13
ZTP-ME	98.75 ± 10.90	99.54 ± 12.41	89.43 ± 8.27

**Table 4:** In vivo pharmacokinetic parameters after administration of ZTP and ZTP-ME.

Parameter	Organ	IV ZTP	IN ZTP	IN ZTP-ME	Oral ZTP- ME
C_max_ (µg/mL)	Plasma	8.18 ± 0.97	2.13 ± 0.14	0.41 ± 0.07	0.34 ± 0.04
	Brain	2.37 ± 0.65	1.90 ± 0.37	4.04 ± 0.26	0.57 ± 0.04
T_max_(h)	Plasma	0.5 ± 0.12	0.5 ± 0.23	1.66 ± 0.65	1± 0.47
	Brain	0.5 ± 0.32	1.02 ± 0.21	0.5 ± 0.26	2.01 ± 0.25
AUC_0–24_ (µg*h/mL)	Plasma	14.27 ±2.49	6.76 ± 0.24	2.98 ± 0.05	2.38 ± 0.71
	Brain	4.40 ± 0.36	5.87 ± 0.47	18.63 ±1.33 ***	3.10 ± 0.92
t_1/2_ (h)	Plasma	2.41 ± 0.96	2.49 ± 0.21	7.94 ±1.82	11.08 ± 1.87
	Brain	0.83 ± 0.21	4.21 ± 1.33	5.31 ± 0.48	8.35 ± 0.29
MRT (h)	Plasma	2.06 ± 0.44	3.956 ± 0.28	12.21 ± 2.91	15.94 ± 2.62
	Brain	0.97 ± 0.15	3.04 ± 0.24	7.99 ± 0.94	14.49 ± 2.38
DTE %	-	-	520.70	3754.97 ****	-
DTP	-	-	80.6	97.54	-

Data are presented as the mean ± S.E.M (*n* = 3). *** *p* < 0.001, a significant difference in AUC_0–t_ of IN ZTP-ME when compared to IV solution and IN ZTP solution. **** *p* < 0.0001; significant improvement in DTE of IN ZTP-ME. IV ZTP: intravenous zotepine; IN ZTP: intranasal zotepine; IN ZTP-ME: intranasal zotepine microemulsion and Oral ZTP-ME: oral zotepine microemulsion.

## Data Availability

Data contained within the article, additional data available from S.R.P., S.S. on reasonable request and responsible for the data provided in the manuscript.

## Supplementary Materials

The following supporting information can be downloaded at: https://www.mdpi.com/article/10.3390/pharmaceutics14050978/s1, Figure S1: Chromatogram of zotepine in (a) Plasma and (b) Brain. Method S1: High-Pressure Liquid Chromatography (HPLC) Analysis.

## Abbreviations

ABC—ATP-binding cassette efflux transporters; CAN—acetonitrile; ATCC—American type culture center; ATR-FTIR—Attenuated total reflectance Fourier transform infrared spectroscopy; BBB—blood–brain barrier; CPCSEA: Committee for the Purpose of Control and Supervision of Experiments on Animals. DMSO—Dimethyl Sulfoxide; DTE %—Drug targeting efficiency %; DTP—Drug transport percentage; EPS—extrapyramidal side effects ; IAEC: Institutional animal Ethical Committee; Intranasal -IN; ME—Microemulsion; MRT—Mean Residence time; MTT—(3-(4,5-dimethylthiazol-2-yl)-2,5-diphenyl tetrazolium bromide); NCCS—National centre for cell science; NS—Nanosuspension; PBS—Phosphate buffer saline; PD—Plain drug; PDI—Polydispersity Index; QbD—Quality by Design approach; Reverse Phase High-Pressure Liquid Chromatography (RP-HPLC); SMEDDS—Self Micro Emulsifying drug delivery systems; SNF—Simulated nasal fluid; TEM—Transmission electron microscopy; ZP—Zeta Potential; ZTP—Zotepine; ZTP-ME-ZTP-loaded microemulsion.
