# Supplementary material for: Brain-Targeted Intranasal Delivery of Zotepine Microemulsion: Pharmacokinetics and Pharmacodynamics
Source: Pharmaceutics. 2022 Apr 30;14(5):978. doi: 10.3390/pharmaceutics14050978 (PMC9145021; doi:10.3390/pharmaceutics14050978)
Supplement: Supplementary file 1 [file pharmaceutics-14-00978-s001.zip › pharmaceutics-1695538-supplementary.pdf]

Supplementary Materials: Brain Targeted Intranasal Delivery of Zotepine Microemulsion: Pharmacokinetics and Pharmacodynamics

Sravanthi Reddy Pailla Sunitha Sampathi, Vijayabhaskarreddy Junnuthula, Sravya Maddukuri, Sujatha Dodoala and Sathish Dyawanapelly

Methods:

S1. High-Pressure Liquid Chromatography (HPLC) Analysis

The analytical method for ZTP was developed using the RP-HPLC system (Shimadzu) equipped with a PDA detector. HPLC analysis was done using C-18 reverse column (Fortis RP-C18) of dimensions (5 µm, 250 mm × 4.6 mm) with ammonium acetate (20 mM): methanol: acetonitrile as mobile phase (10: 45: 45 v/v), at 1000µL/min flow rate. In brief, the drug was dissolved in methanol and acetonitrile (1mg/mL) and a series of standard solutions (0.1, 0.5, 1, 2, 4, 6, 8, and 10 µg/mL) was prepared. Analysis was performed in isocratic mode, a 100 µL sample was injected, and eluents were monitored at a wavelength of 264 nm. The established method was validated for parameters, like accuracy, precision, limit of detection (LOD), limit of quantification (LOQ),linearity, and recovery [1].

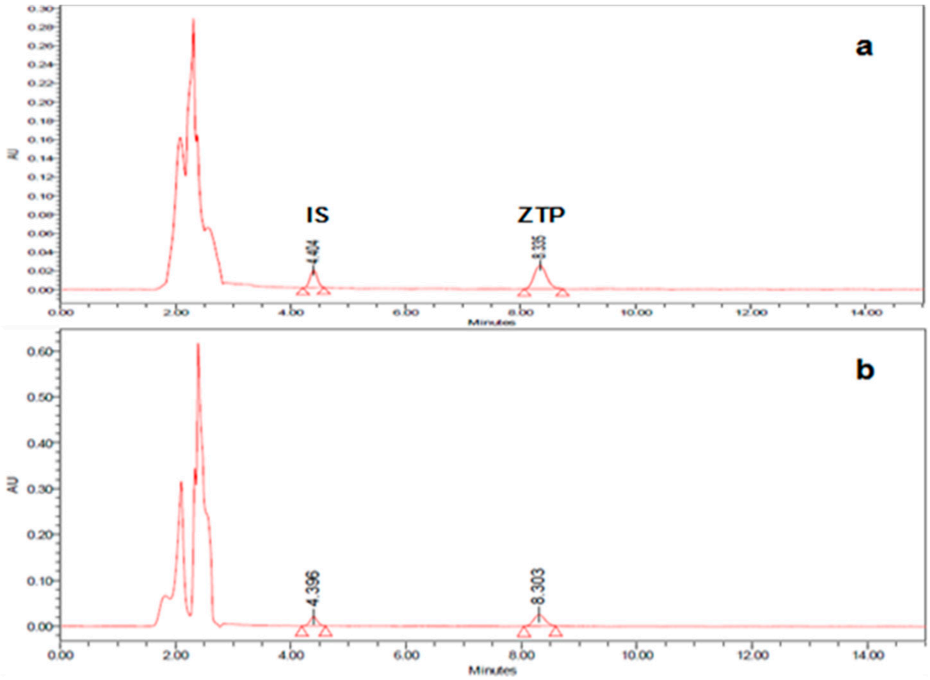

Figure S1. Chromatogram of zotepine in (a) Plasma and (b) Brain.

1. Pailla, S.R.; Talluri, S.; Rangaraj, N.; Ramavath, R.; Challa, V.S.; Doijad, N.; Sampathi, S. Intranasal Zotepine Nanosuspension: Intended for improved brain distribution in rats. *DARU J. Pharm. Sci.* **2019**, *27*, 541–556. https://doi.org/10.1007/s40199-019-00281-4.
